# On the Comparative Influence of the Male and Female Parent upon the Progeny

**Published:** 1859-05

**Authors:** J. B. Thomson

**Affiliations:** Edin., Resident Surgeon, General Prison, Perth


*On the Comparative Influence of the Male and Female Parent upon the Progeny.* By J. B. THOMSON, L. R. C. S., Edin., Resident Surgeon, General Prison, Perth.

The following cases appear to me illustrative of a very curious and not unimportant chapter of anthropology, viz; "The comparative influence of the male and female of the human family upon their progeny"—a subject upon which very crude and indefinite notions are held, not only by the public, but by members of our profession. It is a settled point with many, that it is foolish to search after any laws regulating the transmission of particular textures, features and constitutions from either parent to the offspring.

While it is admitted that we can found little upon mere supposed general physical or psychical resemblance, I think the method of inquiry followed in this paper, is a correct one, and that a number of individual instances of the transmission of abnormal peculiarities from parent to progeny being accumulated and balanced, will lead to a safe and scientific induction.

Mercatus, in his work, "De Morbis Hereditariis," says truly, that the parents, grandparents and great grandparents transmit quality, character, form and structure, proportion and disproportion, or any preternatural condition of a single membrane or organ, part or parts. Of this statement there can be little doubt. We may go further, and affirm that, where we find such irregularities and defects plainly appearing in one parent, and reappearing in any of the offspring, such irregularities or defects are due to the influence of that parent. The order of causation is not to be questioned. And further, when striking abnormal conditions, physical or mental, are transmitted in families, the statistics of such should form data upon which to found a proof whether, and in what proportion, the influence of the male or of the female preponderates. Beginning with physical peculiarities of the external structure, transmitted from parents to progeny, let us examine "the transmission of the skin peculiarities."

Case 1.—Hereditary transmission of webbed fingers.—A. M., Alva, has had a family of 9 children, 5 sons and 4 daughters. He himself and his four daughters are webbed betwixt the middle and ring fingers, or close fingered as their mother calls it, i. e., the skin stretches across and unites these fingers together. None of the sons have this peculiarity. A. M.'s grandfather had the same; also his mother and two sons and one daughter; his uncle, two daughters and one son, this son having all the fingers of both hands webbed together. A. M.'s daughter has one daughter webbed betwixt the middle and ring fingers of both hands.

Case 2.—Hereditary transmission of webbed fingers and toes. (This case, from a recent number of the Lancet, is so similar to the former, that I make no apology for transferring it to this paper, for the sake of illustrating my argument.) W. S. has three fingers united throughout by skin, viz: the middle, the ring, and little fingers of both hands. His mother has the same, but W. S. is the only one of seven children so malformed. Her uncle (her father's brother) had the same, and her paternal grandfather had the three smaller toes on each foot similarly united.

Case 3.—Hereditary transmission of fingers and toes partially webbed.—J. B., Menstrie, has a daughter with six toes on each foot, the little toe and its neighbor being well webbed; also, two little fingers on each hand partially adherent by skin. J. B.'s great grandfather had the same number of toes, and two little fingers on the left hand also partially webbed. No other member of this family can be traced to have had any abnormal physical conformation.

Case 4.—Supernumerary toes and fingers webbed.—J. R., Tillicoultry, has the following peculiarity in his family, viz: one girl webbed betwixt the little toe and its neighbor; one son with two little fingers on each hand and two little toes on each foot. No hereditary trace of these peculiarities can be found in any of the ancestors of this family, unless we admit the account of the mother as the true cause. She says, that when she carried this boy in utero, she met with an accident that split her fingers in two, so that it always afterwards looked like two fingers.

From the small number of cases now set forth it would be unsafe to draw any strong proofs, lest we should be placed in the category of the philosopher in Rasselas, who was always coming to conclusions without anything being concluded. But, although we admit that such a small number of cases is not proof positive, we must allow that they point to the following deductions:

1. That the male parent has a principal share in the transmission of hereditary skin peculiarities to the offspring.

In case 1, we have a grandfather, a father and an uncle sending down an abnormal condition directly through the male line; and a striking resemblance to the male parent belonged to all those descendants who inherit this skin peculiarity. On the other hand, we have a grandmother and a granddaughter transmitting the same directly to their children.

In case 2, the paternal grandfather, and in case 2 the great grandfather, was the original-progenitor, to whom the physical malformations were traced back. Leaving out No. 4, where the origin is very doubtful, we have the following proportional cases, in which the immediate influence of the male exceeds that of the female parents:

| | | | | | |
|---|---|---|---|---|---|
| Case 1.—Transmitted immediately by male, | | | | 10, female, | 4. |
| 2. | " | " | " " | 3, " | 1. |
| 3. | " | " | " " | 2, " | 0. |
| | | | | 15 | 5 |

But these cases point to another interesting deduction.

2. That the skin peculiarity in all these cases, where it could be traced back, had its origin in a male progenitor. In No. 1, it came in with a grandfather; in No. 2 with a paternal grandfather, and in No. 3 with a great grandfather. A curious question here arises: Did the influence of the originator of this malformation extend itself through several generations who bore his peculiar characteristics? Is it true, as Dr. Harvie has recently asserted, that the male is the real producer of the species? Is it true that the influence of the male (in certain nstances) extends beyond the first impregnation?

The consideration of these cases, which show the influence of the male to be greater than that of the female parent in the transmission of skin peculiarities, led me to look at the history of certain skin diseases which are hereditary, and the following instances occurred to my recollection:

Case of the porcupine family.—The original porcupine man, Edward Lambert, had six children and two grandsons, with the same singular skin as himself, resembling, it is said, an innumerable company of warts, of a dark brown color, and a cylindrical figure, rising to an inch in height. In this case, the disease originating in a male, continued to all the family of six, and descended to the grand children.

Leprosy, too, seems to be chiefly derivable from the male parent. In Dr. Simpson's curious inquiries into the history of leprosy, we find quoted from the old Burgh Records of Glasglow (1859,) "Robert Bogell, son to Patrick Bogel," both lepers in that city.

The modern experience of this malady in Norway, where it has so unaccountably increased of late years, has led to serious inquiry how it is to be prevented. Leprosy, or the spedalkshed, is held by Drs. Broek and Danielson to be purely hereditary; and so strong is the opinion of the male being the chief propagator, that the proposal has not only been laid before the Strothing or Norwegian Parliament to prohibit the marriage of a leper, but it has been a topic of public and and professional discussion, how far it would be just to deprive the male infants of leprous parents of the power of propagation.' Ligature of the vasa deferentia, we learn has been seriously contemplated as a national measure.

The analogy of the lower animals confirm these views of the paramount influence of the male in transmiting generally the character of the skin to the progeny. The spawn of the salmon being impregnated with the male trout, the skin and the spots upon it showed the character of the trout, and vice versa, the salmon being the male. With birds, generally, the outer textures follow the male. With quadrupeds, the same rule holds. An intelligent and experienced sheep farmer informs me that it is the practice to cross the blackfaced sheep on the Ochils with the Leicester ram. The Ochil ewes are blackfaced, and have horns. The Leicester ram is not blackfaced and has no horns. The breed follow the Leceistei ram, whitefaced, and in the proportion of about 86 per cent. have no horns. A few years ago, on the estate of Ava, there was a black ram with five horns, two on either side and one on the center. The breed by the common white ewe took the abnormal character of the ram, with a few exceptions, We know also that the products of the male ass by the mare, and of the stallion by she ass, can be distinguished by the skin, having the distinctive characteristics of the sire.

Numerous examples of this law must be well known to cattle dealers, and this subject is admirably treated by Mr. Orton, of Southerland, in his ingenius papers "On the Physiology of Breeding."

We may safely, I think, conclude from the facts before us:

1. That in the lower animals, and in man also, the influence of the male is greater than that of the female parent in the transmission of the skin texture to the progeny.

2. That the exceptional cases (probably more in man than the lower animals) lead us to look for some primary or secondary law presiding over the physiology of generation.

I intend to continue this inquiry as to the influence of the male on the other textures and organs of the body, in a series of cases and notes.— *Virginia Medical Journal.*

